# Comparison of PEEP titration methods to improve respiratory system compliance in acute respiratory distress syndrome: a randomized controlled study

**DOI:** 10.62675/2965-2774.20260143

**Published:** 2026-02-26

**Authors:** Israel Silva Maia, Mariangela Pimentel Pincelli, Cassio Luis Zandonai, Julia Souza de Oliveira, Lucas Tramujas, Juliana Carvalho Ferreira, Alexandre Biasi Cavalcanti

**Affiliations:** 1 Hospital Nereu Ramos Florianópolis SC Brazil Hospital Nereu Ramos - Florianópolis (SC), Brazil.; 2 Research Institute HCor São Paulo SP Brazil Research Institute, HCor-Hospital do Coração - São Paulo (SP), Brazil.; 3 Hospital das Clínicas Faculdade de Medicina Universidade de São Paulo São Paulo SP Brazil Division of Pulmonology, Instituto do Coração, Hospital das Clínicas, Faculdade de Medicina, Universidade de São Paulo - São Paulo (SP), Brazil.

**Keywords:** Respiration, artificial, Respiratory distress syndrome, Positive-pressure respiration, Lung compliance

## Abstract

**Objective:**

To compare the effects of four positive end-expiratory pressure titration methods on respiratory system compliance over the first 3 days of mechanical ventilation and to analyze the agreement between derived positive end-expiratory pressure and compliance values among these methods immediately after randomization.

**Methods:**

Single-center, randomized study acute respiratory distress syndrome patients were assigned to one of four groups based on positive end-expiratory pressure titration methods: electrical impedance tomography, transpulmonary pressure measured via an esophageal catheter, the best compliance approach with daily positive end-expiratory pressure titration, and a control group using a low positive end-expiratory pressure/ fraction of inspired oxygen table with adjustments as necessary. The primary outcome was mean respiratory system compliance over the first 3 days of mechanical ventilation. Immediately post-randomization, the best positive end-expiratory pressure according to each method was assessed for every patient, and within-patient agreement of titrated positive end-expiratory pressure and compliance for pairs of methods was calculated with the Bland-Altman method.

**Results:**

Forty-nine patients participated. Compared to control, the mean difference in compliance was 0.03mL/cmH_2_O (95%CI -2.74 to 2.8) in the Electrical impedance tomography Group; 1.90mL/cmH_2_O (95%CI -0.98 to 4.78) in the Catheter Group, and 1.42mL/cmH_2_O (95%CI -1.35 to 4.19) in the best compliance group. Within-patient agreement of titrated positive end-expiratory pressure and compliance was poor, with 95% limits of agreement ranging from -9.3 to 9cmH_2_O for positive end-expiratory pressure and from -8.5 to 11.4mL/cmH_2_O for compliance.

**Conclusion:**

No significant differences in mean respiratory system compliance were found among positive end-expiratory pressure titration methods compared to control. The agreement between titrated positive end-expiratory pressure and respiratory system compliance using different methods was low.

## INTRODUCTION

Acute respiratory distress syndrome (ARDS) accounts for 10.4% of all intensive care unit (ICU) admissions with a high mortality rate.^(1)^ Lung injury can be aggravated by mechanical ventilation with high tissue stress and strain in a normal ventilated area that is reduced in size due to heterogeneity of lung parenchyma.^(2-4)^ Tidal hyperinflation, even with low lung volume ventilation, may contribute to lung injury as well.^(3,5,6)^ Positive end-expiratory pressure (PEEP) can mitigate this type of lung injury^(5,7)^ by increasing the number of aerated alveoli that participate in current ventilation and improving lung compliance,^(8,9)^ which is inversely related to driving pressure.^(10)^Driving pressure, defined as the ratio of tidal volume (VT) to the respiratory system compliance, serves as an index of the functional size of the lung.^(11)^ High driving pressure is directly related to increased mortality.^(1,10,12)^ Driving pressure can be reduced by lowering VT or increasing compliance, for example, by increasing ventilated areas using adequate PEEP. Ventilation with ultra-low VTs has significantly reduced driving pressure. However, no benefit was found in terms of mortality, and for one-third of patients, it was impossible to attain a VT of 4mL/kg.^(13)^ Furthermore, the association of extracorporeal CO_2_ removal (ECCO_2_R) to facilitate ultra-low VT ventilation did not result in a benefit in mortality as well.^(14,15)^ Conversely, reduction of driving pressure and enhancement of static compliance via PEEP titration may be a valuable strategy to decrease ventilator-induced lung injury. Lower compliance has been associated with higher mortality, although this relationship is nonlinear.^(16,17)^ Nevertheless, analysis of recent studies has shown better outcomes in patients who improved their baseline compliance during the first days of mechanical ventilation.^(16,18)^

There are various methods of PEEP titration, including those based on oxygenation targets, respiratory system compliance, transpulmonary pressure, and electrical impedance tomography (EIT). The oxygenation method employs PEEP/fraction of inspired oxygen (FIO_2_) tables, which offer the benefit of easy integration into clinical practice^(19)^ and is typically used as the control group method in clinical trials.^(20-25)^Optimizing respiratory system compliance may personalize lung mechanics and would presumably result in superior compliance during the first days of mechanical ventilation.^(20,21)^ Positive end-expiratory transpulmonary pressure with an esophageal catheter may avoid alveolar collapse and minimize lung stress.^(26)^ Electrical impedance tomography can bring real-time lung imaging, facilitating the achievement of the best balance between alveolar collapse and overdistention.^(27,28)^However, it remains unclear which of these methods is superior in terms of improving respiratory system compliance and thereby leading to better clinical outcomes.^(12,29)^

Therefore, we compared three PEEP titrating methods applied at the bedside with a control group using a low PEEP/FIO_2_ table to identify the following:

1. Which method best improves respiratory system static compliance (Crs) over the first three days in ARDS patients.
2. The agreement between PEEP settings and respiratory system static compliance values across each pair of methods evaluated for each patient on the first titrating PEEP attempt, right after randomization.

The objective of this study was to compare the effects of four PEEP titration methods on respiratory system compliance over the first 3 days of mechanical ventilation and to analyze the agreement between derived PEEP and compliance values among these methods immediately after randomization.

## METHODS

### Study design

Single-center, ten-bed respiratory medical ICU located in Florianópolis (SC), Brazil. Randomized clinical trial comparing three different methods of PEEP titration - EIT, transpulmonary pressure measured with the esophageal catheter, and best compliance to a control group with low PEEP/FiO_2_ table in patients with ARDS. The institutional research ethics committee approved the trial with the approval number: 73057317.8.1001.0115. Date: December 21^st^, 2017. A next of kin signed the informed consent form before the participant enrollment.

### Patients

Eligible patients were those who met ARDS criteria^(30)^ for less than 36 hours. PaO_2_/FiO_2_ criterion was confirmed after one hour of standardized ventilation according to the ARDSNet protocol (VT ≤ 6mL/kg of predicted body weight, plateau pressure < 30cmH_2_O, and PEEP titrated according to the low PEEP/FiO_2_ table).^(31)^ Baseline values, including respiratory system compliance, were recorded at the time eligibility criteria were being assessed. Exclusion criteria and details on the standardized ventilation settings are provided in Sections 1S and 2S (Supplementary Material).

### Randomization

Randomization was stratified according to compliance (lower than or equal to 30 or higher than 30mL/cmH_2_O) and assessed electronically at https://www.sealedenvelope.com. Concealment of the allocation list was ensured through the central randomization system. Patients were allocated following a 1:1:1:1 ratio.

### Blinding

There was no blinding of investigators, participants, and outcome assessors.

### Interventions

#### Preparation for all patients after randomization

Patients were fully sedated and received neuromuscular blockade while in the supine position, with the head of the bed elevated to 30^o^. All patients received an esophageal balloon catheter and an EIT belt at this time. Mechanical ventilation settings for PEEP titration are provided in Section 3S (Supplementary Material).

#### Positive end-expiratory pressure titration immediately after randomization (Day 0)

**Figure 1:**
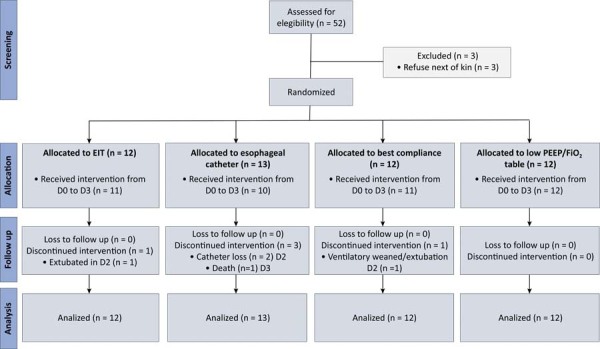
Flow of participants according to the Consolidated Standards of Reporting Trials diagram from screening to analysis.

**Figure 2:**
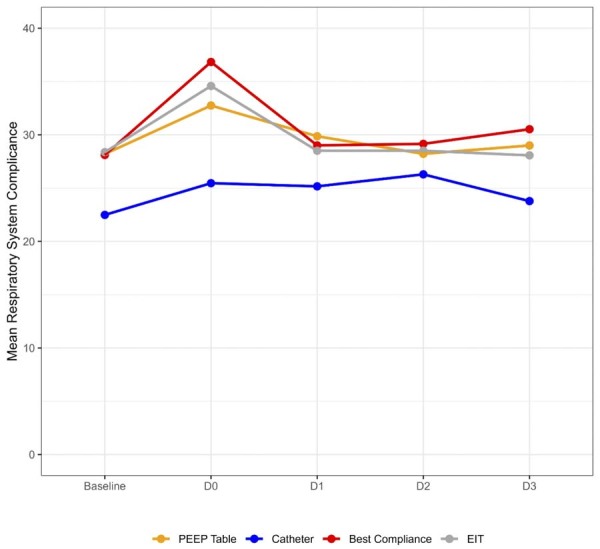
Mean respiratory system compliance evolution from baseline through Day 3.

**Figure 3:**
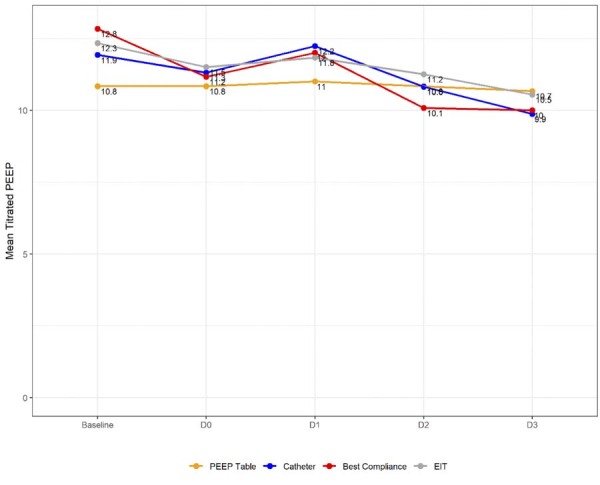
Mean positive end-expiratory pressure values from baseline through Day 3.

**Figure 4:**
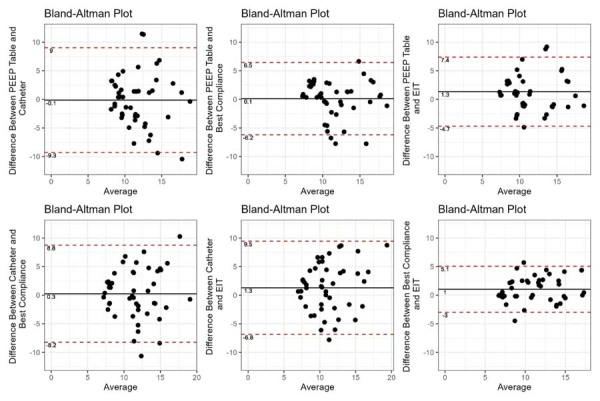
Mean differences of positive end expiratory pressure values between positive end expiratory pressure titration methods, in addition to 95% limits of agreement.

Positive end-expiratory pressure was titrated using all four methods at the same time (Figure 1S - Supplementary Material): EIT, transpulmonary pressure with an esophageal catheter, best compliance, and low PEEP/FiO_2_. Initially, PEEP was titrated according to the low PEEP/FiO_2_ table (Table 1S - Supplementary Material) to maintain SpO_2_ between 90 - 96%. Thereafter, PEEP was increased in steps of 2cmH_2_O every 30 seconds until a maximum of 23cmH_2_O, when a decremental PEEP maneuver was initiated until a minimum of 7cmH_2_O. Titrated PEEP was considered according to the corresponding intercept point of cumulative collapse and overdistension curves for the EIT Group, transpulmonary expiratory pressure > 0 and ≤ 2cmH_2_O for the esophageal Catheter Group, and pressure measured immediately before the decrease ≥ 1cmH_2_O in C_rs_ in the best compliance group. More details in Section 4S, Figures 2S, 3S, and 4S (Supplementary Material). After determining the titrated PEEP using all individual methods for each patient, PEEP was set according to the assigned group and maintained until the next morning, except for the control group, whose settings were dynamically adjusted as needed during the 24 hours in accordance with the low PEEP/FiO_2_ table.

#### Positive end-expiratory pressure titration from Day 1 to Day 3

Positive end-expiratory pressure was titrated daily exclusively according to the randomly assigned method and maintained until the next morning, except in the control group, as explained above. Tidal volume was set between 4 and 6mL/kg, and plateau pressure was lower than 30cmH_2_O. No recruitment maneuver was performed immediately after PEEP titration, as per the protocol. All patients under invasive mechanical ventilation used a closed suction system until extubation, minimizing disconnections and reducing complications. The healthcare team was trained to perform suctioning only when clinically indicated, based on specific signs and symptoms. In the event of accidental disconnection, PEEP titration was repeated according to the patient’s assigned allocation group, ensuring consistency with the study design and individual requirements. Details are described in Section 5S (Supplementary Material).

## Data collection and outcomes

Data were collected immediately before randomization (baseline), during the first PEEP titration maneuver immediately after randomization (D0), 1 hour after PEEP titration on D0, and then, every morning, starting on the day after randomization (Day 1) up to Day 3, at ICU discharge or hospital discharge, and on Day 28. Details of data collection are specified in Section 6S (Supplementary Material).

The primary outcome was mean respiratory system compliance (C_rs_) from Day 0 to Day 3. Exploratory outcomes are described in Section 7S (Supplementary Material).

## Statistical analysis

The calculated sample size was 15 patients in each group, resulting in a total of 60 patients in the final sample. With this sample size, the study would have 80% power to detect a compliance difference of 5mL/cmH_2_O between groups, considering an alpha error of 5%, analysis of covariance (Ancova), standard deviation of 8mL/cmH_2_O, and a Pearson’s r of 0.8 between baseline compliance and mean compliance on Days 0 to 3.

Numerical variables were described as median and interquartile range (IQR), and compared between groups using the Kruskal-Wallis or Mann-Whitney Wilcoxon test. Categorical variables were described in terms of absolute and relative frequencies and compared at each time point using Fisher’s exact test.

Values of titrated PEEP on Day 0 according to pairs of PEEP titration methods were compared using Pearson’s correlation coefficient, and agreement was assessed using Bland-Altman analysis. Similarly, values of C_rs_ corresponding to PEEP titrated according to pairs of methods were also analyzed with Pearson’s correlation and Bland-Altman analysis.

The effects of the PEEP titration method on the primary outcome were assessed using a mixed linear regression model adjusted for the treatment group and baseline compliance. For a *post hoc* sensitivity analysis, we used an interaction term between treatment group and time. Results for the models were presented as absolute differences, 95% confidence intervals (95%CIs), and p-values. The significance level was 0.05. There was no p-value adjustment for multiple comparisons. There were no interim analyses. All analyses were performed using the R software.^(32)^

## RESULTS

### Patients

From April 2018 to May 2021, 52 patients diagnosed with ARDS were screened, with 49 patients included and randomized to the 4 treatment groups: 12 to the EIT Group, 13 to the esophageal Catheter Group, 12 to the best C_rs_ group, and 12 to the PEEP/FiO_2_ table group. All randomized patients were followed for 28 days and included in the final analysis (Figure 1). The study was stopped before reaching the predicted sample size due to a low recruitment rate and limited funding.

EIT - electrical impedance tomography; PEEP - positive end expiratory pressure; FiO_2_ - fraction of inspired oxygen. Full intervention: positive end expiratory pressure titration according to the allocated method on all 3 study days. Partial intervention: positive end expiratory pressure titration following the allocation was not applied to all 3 days. The reason for the discontinuation is indicated in the line below each item within the follow-up boxes.

Demographic characteristics at baseline were generally comparable across the four groups; however, participants in the EIT and esophageal catheter groups were older (Table 1). The median age was 47 years (IQR 38 - 55), with a Simplified Acute Physiology Score 3 (SAPS 3) of 48 points (IQR 43 - 54); 59% were men. The main comorbidity was human immunodeficiency virus (HIV) infection in 35%, followed by obesity in 22% and hypertension in 20%. All 49 participants had ARDS of pulmonary origin, 59% of which were secondary to severe community-acquired pneumonia.

**Table 1:** Baseline characteristics according to treatment group

Variable	EIT (n = 12)	Catheter (n = 13)	Best compliance (n = 12)	PEEP table (n = 12)	Total (n = 49)
Sex					
Female	5/12 (42)	7/13 (54)	3/12 (25)	5/12 (42)	20/49 (41)
Male	7/12 (58)	6/13 (46)	9/12 (75)	7/12 (58)	29/49 (59)
Age	50 (45 - 64)	47 (42 - 55)	44 (3 - 49)	42 (32 - 56)	47 (38 - 55)
Comorbidities					
HIV	3/12 (25)	2/13 (15)	7/12 (58)	5/12 (42)	17/49 (35)
Obesity	4/12 (33)	3/13 (23)	2/12 (17)	2/12 (17)	11/49 (22)
Arterial hypertension	5/12 (42)	1/13 (8)	1/12 (8)	3/12 (25)	10/49 (20)
Diabetes	3/12 (25)	1/13 (8)	2/12 (17)	2/12 (17)	8/49 (16)
Chronic renal failure	0/12 (0)	1/13 (8)	0/12 (0)	0/12 (0)	1/49 (2)
COPD	1/12 (8)	0/13 (0)	0/12 (0)	0/12 (0)	1/49 (2)
Others	4/12 (33)	6/13 (46)	2/12 (17)	5/12 (42)	17/49 (35)
Pulmonary ARDS	12/12 (100)	13/13 (100)	12/12 (100)	12/12 (100)	49/49 (100)
Community-acquired pneumonia	8/12 (67)	7/13 (54)	8/12 (67)	6/12 (50)	29/49 (59)
COVID-19	3/12 (25)	2/13 (15)	2/12 (17)	1/12 (8)	8/49 (16)
H1N1	0/12 (0)	2/13 (15)	0/12 (0)	2/12 (17)	4/49 (8)
Influenza A	0/12 (0)	0/13 (0)	1/12 (8)	1/12 (8)	2/49 (4)
Bronchoaspiration	0/12 (0)	1/13 (8)	0/12 (0)	1/12 (8)	2/49 (4)
SAPS 3	51 (47 - 59)	45 (41 - 50)	48 (44 - 55)	47 (38 - 54)	48 (43 - 54)
PaO_2_/FiO_2_	129 (77 - 182)	165 (105 - 196)	190 (152 - 211)	192 (169 - 219)	172 (117 - 203)
PBW	59 (57 - 62)	59 (53 - 66)	64 (58 - 74)	66 (57 - 72)	61 (55 - 67)
Tidal volume; median/kg pbw	5.98 (5.95 - 6.09)	6 (5.89 - 6.1)	5.94 (5.08 - 6.01)	6.01 (5.95 - 6.03)	6.00 (5.89 - 6.06)
PEEP	12 (10 - 14)	10 (10 - 14)	11 (10 - 15)	10 (10 - 10.5)	10 (10 - 14)
Pplat	25 (23 - 29)	27 (26 - 30)	29 (26 - 30)	24 (23 - 27)	26 (23 - 30)
Driving pressure	13 (11 - 14)	16 (15 - 18)	15 (12 - 19)	13 (12 - 16)	14 (12 - 16)
FiO_2_	85 (68 - 100)	70 (60 - 80)	70 (60 - 70)	70 (60 - 80)	70 (60 - 80)
Crs	26 (25 - 33)	22 (18 - 25)	25 (21 - 29)	26 (23 - 33)	25 (22 - 32)
Inspiratory transpulmonary pressure	13.1 (9.1 - 15) (n = 10)	15.1 (13.6 - 16.15) (n = 11)	11.9 (10.4 - 16) (n = 9)	10.4 (8.1 - 14.2) (n = 12)	13.2 (10.2 - 15.9) (n = 42)
Expiratory transpulmonary pressure	0.4 (-1.9 - 1.9) (n = 10)	1.0 (0.3 - 2.5) (n = 11)	1.8 (-0.5 - 3.9) (n = 9)	-0.2 (-3.3 - 1.0) (n = 12)	0.8 (-1.2 - 2.6) (n = 42)

EIT - electrical impedance tomography; PEEP - positive pressure ventilation; HIV - human immunodeficiency virus; COPD - chronic obstructive pulmonary disease; ARDS - acute respiratory distress syndrome; COVID-19 - coronavirus disease 2019; SAPS 3 - Simplified Acute Prediction Score 3; PaO_2_ - arterial partial pressure of oxygen; FiO_2_ - inspired fraction of oxygen; Pplat - plateau pressure; Crs - compliance respiratory system; Pbw: predicted body weight according to formulas: (height cm - 152) X 0.91 + 50 (men) and (height cm - 152 ) x 0.91 + 45.5. Results expressed as n/N (%) or median (interquartile range).

Ventilatory and blood gas variables were also adequately balanced between the groups at baseline, except for those in the EIT Group who had a lower PaO₂/FiO₂ ratio, higher PaCO_2_ and PaO_2_/FiO_2_ ratio in the catheter treatment group and PEEP table group, respectively, compared to the other groups as shown in table 1 and table 2S (Supplementary Material). Most patients had moderate ARDS with a median PaO_2_/FiO_2_ ratio of 172mmHg (IQR 117 - 203), PEEP of 10cmH_2_O (IQR 10 - 14), compliance of 25mL/cmH_2_O (IQR 22 - 32), transpulmonary inspiratory pressure of 13.2cmH_2_O (IQR 10.2 - 15.9), and transpulmonary expiratory pressure of 0.8cmH_2_O (IQR -1.2 - 2.6). The median of these individualized values per group during the 3 days of intervention are specified in tables 2S and 3S (Supplementary Material). There were no differences between groups in the median titrated PEEP values over time from Day 0 to Day 3.

**Figure 5:**
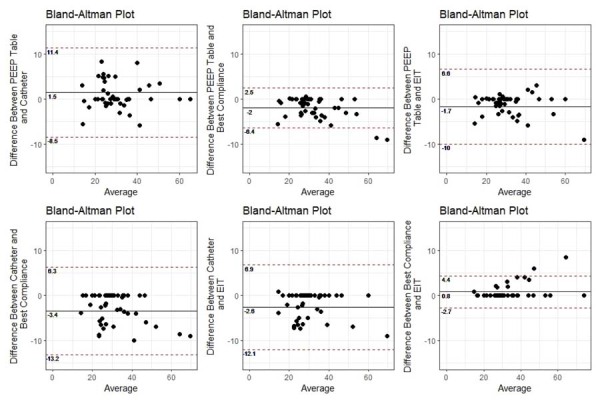
Mean differences of respiratory system compliance values between positive end expiratory pressure titration methods in addition to 95% limits of agreement. PEEP - positive end expiratory pressure; EIT - electrical impedance tomography.

The mean respiratory system compliance on Day 0 was 29.5 (8.9) mL/cmH_2_O in the EIT Group, 24.9 (7.3) mL/cmH_2_O in the Catheter Group, 30.0 (12.6) mL/cmH_2_O in the best compliance group, and 29.9 (7.6) mL/cmH_2_O in the PEEP/table group (Figure 2). Mean C_rs_ over three days increased in all groups, with greater variation in the Catheter Group, which started and ended with lower values compared to the other groups. Significant variability was observed in the individually titrated PEEP and C_rs_ values, as well as in the mean titrated PEEP for each group over the course of three days (Figure 3 and Figures 5S and 6S [Supplementary Material]).

PEEP - positive end expiratory pressure, EIT - electrical impedance tomography.

PEEP - positive end expiratory pressure, EIT - electrical impedance tomography.

### Primary outcome

As compared to the low PEEP/FIO_2_ table, the change in mean C_rs_ from Day 0 to Day 3 adjusted by the baseline compliance was 0.03mL/cmH_2_O (95%CI -2.74 - 2.8; p = 0.98) in the EIT titration group, 1.9mL/cmH_2_O (95%CI -0.98 - 4.78; p = 0.20) in the esophageal catheter titration group, and 1.42mL/cmH_2_O (95%CI -1.35 - 4.19; p = 0.31) in the best C_rs_ group. There were no significant differences in mean C_rs_ between the methods in the first 3 days of PEEP titration (Table 2, Figure 2). Baseline respiratory system compliance was significantly associated with mean C_rs_ over the first three days, with an estimated β coefficient of 1.05mL/cmH_2_O (95%CI 0.93 - 1.16; p < 0,01).

**Table 2:** Primary outcome analysis from Day 0 through Day 3

	Estimate	SE	95%CI	p value
(Intercept)	0.53	1.96	(-3.29 - 4.35)	0.22
Group EIT	0.03	1.45	(-2.74 - 2.8)	0.98
Group catheter	1.90	1.39	(-0.98 - 4.78)	0.20
Group best compliance	1.42	1.39	(-1.35 - 4.19)	0.31
Baseline compliance	1.05	0.06	(0.93 - 1.16)	< 0.01

SE - standard error, 95%CI - 95% confidence interval; EIT - electrical impedance tomography.

The Day 0 median titrated PEEP in the EIT Group was 11 (IQR 7 - 13), 11 (IQR 9 - 13) in the Catheter Group, 11 (IQR 9 - 13) in the best compliance group, and 10 (IQR 10 - 14) in the PEEP/FiO_2_ table group (Table 3S - Supplementary Material). The correlation between PEEP titrated by the four methods varied considerably from a strong correlation between the PEEP titrated with the best respiratory system compliance method and PEEP titrated with the EIT method (r = 0.84; p < 0.001) (Figure 7S - Supplementary Material), moderate correlation between the PEEP titrated by the PEEP/FiO_2_ table with PEEP titrated with the EIT (r = 0.54; p < 0.001) and with PEEP titrated by the best respiratory system compliance method (r = 0.58; p < 0.001), and weak correlation between PEEP titrated with the esophageal catheter method and all other methods (r = 0.34 for EIT; p < 0.05; 0.33 for the best compliance method; p < 0.05 and 0.13 for the PEEP/FiO_2_ table). There was a strong correlation between all methods related to the value of Crs calculated from the titrated PEEP values on Day 0 (Figure 8S - Supplementary Material).

The mean difference between the pairs of titrated PEEP values obtained by the different methods was small, varying from -0.1cmH_2_0 between the PEEP-FiO_2_ table and the esophageal catheter method to 1.3cmH_2_0 between the PEEP-FiO_2_ table and the EIT and esophageal catheter methods. However, the agreement between them was low, with 95% limits of agreement varying from -9.3 to 9.0cmH_2_O between the PEEP-FiO_2_ table and esophageal catheter method to -3.0 to 5.1cmH_2_O between the best compliance method and EIT (Figure 4). The mean difference of C_rs_ values derived from the titrated PEEP was slightly higher than the mean difference of PEEP values, varying from -3.4mL/cmH_2_O between the esophageal catheter and the best compliance method to 1.5mL/cmH_2_O between the PEEP-FiO_2_ table and the esophageal catheter. Nevertheless, the agreement of these methods for C_rs_ values was also low, with 95% limits of agreement varying from -8.5 to 11.4mL/cmH_2_O between the PEEP-FiO_2_ table and the esophageal catheter, and from -2.7 to 4.4mL/cmH_2_O between the best compliance method and EIT (Figure 5). Details are explained in Section 8S (Supplementary Material).

PEEP - positive end expiratory pressure; EIT - electrical impedance tomography.

### Exploratory outcomes

There was no significant difference between groups regarding exploratory outcomes (Table 4S - Supplementary Material). Median ICU and hospital length of stay were 12 (IQR 8 - 18) and 18 (IQR 11 - 23), respectively. 28-day mortality was 39%, and 49% were submitted to the prone position. None of the patients had barotrauma.

A sensitivity analysis for the primary outcome revealed no significant difference in mean compliance from Day 0 to Day 3 between the three methods of PEEP titration and the control (Table 5S - Supplementary Material).

## DISCUSSION

In this study, patients with ARDS were randomized to PEEP titration using EIT, an esophageal catheter, best compliance, and a low PEEP/FiO_2_ table. The compliance levels between the PEEP titrating methods in the first three days were not different after adjusting for the baseline compliance. Correlation of titrated PEEP values between pairs of methods was variable; however, it was strong regarding C_rs_ values generated by the titrated PEEP. In the pairwise comparison between methods, mean PEEP and C_rs_ differences were minor, although with poor agreement.

The four different PEEP titration methods discussed in this manuscript target different aspects of lung mechanics and gas exchange. The PEEP/FiO_2_ table, derived from ARDSNet trials,^(19,31)^ provides a standardized, non-individualized approach aimed at ensuring adequate oxygenation. Nevertheless, this strategy relies primarily on oxygenation as a surrogate marker, which can be misleading. Arterial oxygenation is not solely dependent on alveolar recruitment; it is also significantly influenced by hemodynamic factors. Therefore, following such tables may not always be appropriate in clinical practice, as some patients with severe hypoxemia may show little or no improvement in oxygenation despite higher PEEP levels.^(12)^ In contrast, best compliance titration seeks the PEEP level associated with maximal respiratory system compliance, assuming that optimal alveolar recruitment occurs at this point. However, compliance can be paradoxically improved by intra-tidal recruitment-derecruitment, which can confound interpretation.^(29)^ In addition, since respiratory system compliance reflects both lung and chest wall mechanics, observed changes may result from chest wall alterations rather than proper lung recruitment. Moreover, as PEEP increases, simultaneous recruitment and overdistention in different lung regions can offset each other, leading to minimal or even increased overall compliance despite overdistention of nondependent areas of the lung.^(29)^ Esophageal manometry uses measurements of esophageal pressure to assess transpulmonary pressure, which is used to estimate the alveolar distending force, guiding PEEP adjustments to prevent alveolar collapse, aiming for expiratory transpulmonary pressure higher than or equal to zero. Electrical impedance tomography enables real-time visualization of regional ventilation, generating maps that distinguish areas of collapse, regular aeration, and overdistension.^(29)^ This allows for individualized PEEP titration aimed at maximizing alveolar recruitment while minimizing regional overdistension. Each method targets a different physiological aspect – oxygenation, mechanical efficiency, lung protection, or regional recruitment – and its clinical applicability varies depending on the available resources and patient phenotype. Chiumello et al. evaluated four bedside PEEP titration methods to determine which method would provide the optimal PEEP levels in relation to lung recruitability and ARDS severity.^(33)^ Lung compliance and PEEP values were similar between the methods, as was the case in our study. The only methods that correlated with each other were those generated by the Express study methods^(22)^ and the stress index method,^(34)^ whose choice of PEEP is based on lung mechanics. This also occurred in our study, where we found a strong correlation between the EIT and the best compliance method, as they both aim to minimize lung collapse and overdistension.^(27)^ The lack of correlation between the PEEP titration method using the esophageal catheter and the PEEP/FiO_2_ table in our study can be explained by the fact that oxygenation is the target of the latter approach. Nonetheless, the same level of oxygenation can be achieved in non-aerated lung areas, which vary from 20 to 40% of the entire lung.^(35)^ The esophageal catheter titration method seeks a more open lung through an expiratory transpulmonary pressure other than zero, to prevent alveolar collapse.^(36)^ This is antagonistic to the rationale in the PEEP/FiO_2_ table.

The strong correlation between the methods for measuring lung compliance in this study can be explained by three reasons: first, the lack of precision in the method of measuring Crs, as it was performed manually at the bedside with the ventilator display parameters for all methods. Second, different levels of PEEP in the titration maneuver on the descending curve showed equal values of compliance. Third, due to the probable situation of compliance values in the linear portion of the Pressure x Volume curve of the respiratory system. Different PEEPs can determine different functional residual capacities along this same linearity, which changes more significantly in the initial and final portions of the curve and can show more similar values in the straighter area.^(37)^ The minimal mean difference in Crs values and the highest agreement of these values were observed between the best compliance-guided method and EIT. Our study found that Crs estimates derived from the best compliance method consistently exceeded those obtained via EIT. Electrical impedance tomography calculates a weighted sum of pixel values, enabling the assessment of the total lung collapse and hyperinflation at each PEEP level to determine the most balanced compromise between global hyperinflation and collapse.^(28)^ Nevertheless, there is an enhancement of aerated lung areas, which correlates with an increase in respiratory system compliance, achieving maximal values that align with expectations from the best compliance-guided method. This supports our observation of concordance between the two methodologies.

Several clinical trials compared different PEEP titration methods with the PEEP/FiO_2_ table. These methods were done according to the best compliance of the respiratory system, with or without recruitment maneuver, to the transpulmonary pressure (P_L_) table and to EIT^(20-25,38,39)^in participants with ARDS. Cavalcanti et al.^(24)^ demonstrated a significant difference in PEEP titration between the groups during the first three days of mechanical ventilation. Talmor et al. also showed a significant difference between the titrated PEEP and lung compliance between the groups in the first three days.^(23)^ Costa et al.^(39)^ found a difference in PEEP values between the groups in only 59% of the patients. This was not observed in the present study in any of the methods employed. Nevertheless, the definition of selected PEEP in the study by Costa et al.^(39)^ differed from ours. In that study, PEEP was set at the level corresponding to equal amounts of collapse and overdistension, as estimated by EIT. In contrast, our study defined PEEP as the value corresponding to the intercept point of the cumulative percentage curves of collapse and overdistension. This methodological difference may help explain the divergent findings between the two studies. The compliance of the respiratory system in the study by Cavalcanti et al.^(24)^ was significantly different between the groups on the first day; however, it became similar on the third day, as was also found in our study. Pintado et al.^(20)^ and Beitler et al.^(25)^ found no significant difference in PEEP titration between groups in the first three days of mechanical ventilation, as in the present study. There was no significant difference in lung compliance between the groups in the first week of treatment in the study by Pintado et al.,^(20)^ which is in line with our findings. In contrast to our results, Talmor et al.^(23)^ demonstrated a substantial improvement in compliance—approximately 10mL/cmH₂O – in the esophageal pressure–guided group. Similarly, Kacmarek et al.^(21)^ and Cavalcanti et al.^(24)^ reported clinically meaningful compliance improvements in groups receiving PEEP titrated by the best compliance method. Additionally, Costa et al.^(39)^ showed a sustained compliance gain of about 5mL/cmH₂O in the EIT-guided group. However, these studies shared a critical methodological distinction from ours: all employed aggressive recruitment maneuvers prior to PEEP titration to maximize alveolar recruitment and lung homogenization before determining optimal PEEP. This pre-titration lung recruitment likely influenced subsequent compliance values and PEEP selection. In contrast, our study intentionally avoided recruitment maneuvers due to safety concerns highlighted in the study by Cavalcanti et al.^(24)^ which demonstrated increased mortality and barotrauma in the group receiving recruitment maneuvers and PEEP titration guided by best compliance. This design choice may have contributed to the more modest and transient compliance effects seen in our study. Besides, PEEP levels were set to achieve an expiratory transpulmonary pressure of 0 to 10cmH_2_O in the Talmor et al. study,^(23)^ which contrasts with the present study, which limited PEEP levels to achieve a lower expiratory transpulmonary pressure of 0 to 2cmH_2_O. He et al.^(38)^ found no significant difference between the methods of the titrated PEEP value or lung compliance values in the first 3 days of mechanical ventilation, in line with our findings.

Our study suggests that there is no distinguishable advantage in favor of one method over another. The findings of our study can be interpreted as evidence that there are no real differences between the impact of the four tested methods of titrating PEEP on respiratory system compliance over time. Although a recent study found no association between compliance and mortality in ARDS patients,^(10)^ a large epidemiological study revealed that a decrease in C_RS_ on the first day of ARDS was independently associated with higher mortality.^(16)^ The randomized controlled nature of our study provides a high level of scientific rigor, which facilitates the establishment of a clear causal inference between the intervention under investigation and the resultant outcomes. However, randomized clinical trials provide estimates of the mean treatment effect, which can vary significantly between individuals.^(40,41)^ Furthermore, the great variability of the recruitment potential of the lung in ARDS can be responsible for the heterogeneity of the PEEP effect^(42)^ as well as the difference in the pathophysiological approach used to explain the rationale for each titration method tested, ranging from the best oxygenation target used in the PEEP/FiO_2_ table associated with larger areas of collapse to methods that prioritize better lung mechanics such as EIT and best compliance method targeting a lung with fewer areas of collapse. Notwithstanding, even randomized controlled trials that showed differences in PEEP, driving pressure, VT, and compliance values between groups failed to show a difference in mortality in favor of the intervention group.^(13,14,20,23-25)^

An important consideration in interpreting our results is the potential heterogeneity of treatment response to PEEP titration strategies among ARDS patients. The absence of statistically significant differences in mean respiratory system compliance across study arms does not preclude meaningful variability in individual responses. It remains plausible that certain patients may benefit disproportionately from one titration strategy over another—for example, a compliance-guided approach may yield the most physiologically appropriate PEEP for one individual, whereas an esophageal pressure–guided strategy may be optimal for another. A natural hypothesis is that the value of baseline compliance achieved with each technique might predict the individual effect of different PEEP titration techniques on follow-up respiratory system compliance. Some insight into that hypothesis is provided by observing what happened with the best-compliance approach group the alternative titration methods. Nevertheless, subsequent 3-day respiratory system compliance in this group, who had PEEP titrated daily using the best compliance technique, was not superior to that of the groups using other PEEP titration techniques. This observation provides evidence that challenges the hypothesis that one can reliably select the optimal PEEP titration technique based on respiratory system compliance baseline variables alone. Nevertheless, there may still be room to explore other methods for predicting the individualized effect of PEEP titration techniques on follow-up respiratory system compliance.

Our study has limitations. The pre-specified sample size was not reached due to the low patient recruitment rate and constraints in available financial resources. Nevertheless, the upper limits of 95%CIs for comparisons of all groups with the low PEEP/FIO_2_ table exclude a difference in respiratory system static compliance larger than 5mL/cmH_2_O. Although randomized, the study was not blinded, which may also contribute to performance bias. The small sample size did not allow us to perform subgroup analysis. The study was performed in a single center specialized in infectious diseases, contributing to the high number of HIV cases and pulmonary ARDS in our sample, which may reduce the generalizability of the findings.

On the other hand, to the best of our knowledge, no other study has compared these four methods simultaneously in a randomized manner, ensuring greater control over confounding factors and thereby increasing the reliability of the results. Moreover, we acknowledge that using a target SpO₂ range of 90 - 96% may allow some variability in FiO₂ adjustments between patients, which could, in turn, influence the selected PEEP when using the PEEP/FiO₂ table. However, this range reflected current best practice^(43)^ and was applied uniformly as a clinical target across groups of participants. Besides, although balloon volume was not titrated after prone positioning and the adequacy of standard end-expiratory transpulmonary pressure targets (zero to +2cmH₂O) in the prone position remains uncertain, we mitigated this limitation by verifying catheter position daily using standardized maneuvers, consistently performed by the same physician–physiotherapist team.

## CONCLUSION

In conclusion, no significant differences were observed between the four methods of titrating PEEP regarding lung compliance during the first three days of mechanical ventilation in patients with acute respiratory distress syndrome, suggesting that there is no significant advantage of one method over another in terms of the mean treatment effect. This study has implications for both research and clinical practice. Assuming the critical mediator of ventilator-induced lung injury is driving pressure, which is intrinsically linked to compliance, our findings demonstrate no advantage of one method of PEEP titration over another. Therefore, it seems unlikely that future research comparing different PEEP titration methodologies will achieve substantial differences in respiratory compliance or driving pressure. For clinical practice, our results suggest that preference should be given to the most straightforward, economical, and least invasive technique, that is, the use of the PEEP/FiO_2_ table.

## SUPPLEMENTARY MATERIAL

Supplementary material 1
