# Supplementary material for: Comparison of PEEP titration methods to improve respiratory system compliance in acute respiratory distress syndrome: a randomized controlled study
Source: Crit Care Sci. 2026 Feb 26;38:e20260143. doi: 10.62675/2965-2774.20260143 (PMC13124104; doi:10.62675/2965-2774.20260143)
Supplement: Supplementary material 1 [file 2965-2774-ccsci-38-e20260143-suppl01.pdf]

*Critical Care Science*
https://doi.org/10.62675/2965-2774.20260143

SUPPLEMENTARY MATERIAL

# Comparison of PEEP titration methods to improve respiratory system compliance in acute respiratory distress syndrome: a randomized controlled study

Israel Silva Maia[1], Mariangela Pimentel Pincelli[1], Cassio Luis Zandonai[1], Julia Souza de Oliveira[2], Lucas Tramujas[2], Juliana Carvalho Ferreira[3], Alexandre Biasi Cavalcanti[2]

## SECTION 1S - STANDARDIZED VENTILATION SETTINGS FOR ELIGIBILITY EXCLUSION CRITERIA

Potentially eligible patients were ventilated according to a low PEEP/$FiO_2$ table for 1 hour of standardized ventilation according to the ARDSNet protocol (tidal volume ≤ 6mL/kg of predicted body weight, plateau pressure < 30cm$H_2O$ and PEEP titrated according to low PEEP/$FiO_2$ table.[(1)] Subsequently, all baseline parameters and an arterial blood gas sample were collected. If the ratio of arterial oxygen partial pressure and inspired fraction of oxygen (PEEP/$FiO_2$) remained lower or equal to 300, patients were considered eligible.

## SECTION 2S - EXCLUSION CRITERIA

Patients younger than 18 years old, with intracranial hypertension, acute neurological disease, or the presence of bronchopleural fistula or barotrauma were excluded.

## SECTION 3S - INITIAL MECHANICAL VENTILATION SETTINGS AFTER RANDOMIZATION FOR PEEP TITRATION

Mechanical ventilation was set in volume-controlled mode, $FiO_2$ 100%, respiratory rate 20 bpm, flow 30 to 60L/minute, target tidal volume was 6mL/kg predicted body weight or lower (4 - 5mL/kg) to maintain plateau pressure lower than 30cm$H_2O$.

## SECTION 4S - PEEP TITRATION RIGHT AFTER RANDOMIZATION (D0)

Initially, PEEP was titrated according to the low PEEP/$FiO_2$ table (Table 1S) with the PEEP/$FiO_2$ combination to maintain $SpO_2$ between 90 - 96%. After titrating PEEP by this first method, PEEP was increased in steps of 2cm$H_2O$ each 30 seconds until a maximum of 23cm$H_2O$. Safety criteria for stopping this maneuver were: systolic blood pressure lower than 90mmHg, diastolic blood pressure lower than 65 mmHg, cardiac arrhythmias as and $SpO_2$ lower to 88% the procedure was stopped and only tried again if patient's clinical condition stabilized.

**Table 1S -** Low positive pressure ventilation inspired fraction of oxygen table. Combinations of inspired fraction of oxygen and positive pressure ventilation to maintain oxygen saturation between 90 - 96%

| $FiO_2$ | 30% | 40% | 40% | 50% | 50% | 60% | 70% | 70% | 70% | 80% | 90% | 90% | 90% | 100% |
|---|---|---|---|---|---|---|---|---|---|---|---|---|---|---|
| PEEP | 5 | 5 | 8 | 8 | 10 | 10 | 10 | 12 | 14 | 14 | 14 | 16 | 18 | 18 - 24 |

$FiO_2$ - inspired fraction of oxygen; PEEP - positive pressure ventilation.



This is an open access article under the CC BY license (https://creativecommons.org/licenses/by/4.0/).

After achieving the maximum PEEP of 23cm$H_2O$, a decremental PEEP maneuver was started in steps of 2cm$H_2O$ each minute until the minimum of 7cm$H_2O$ was reached. After determining the titrated PEEP using all individual methods for each patient, PEEP was set according to the assigned group and maintained until the next morning, except for the control group, whose settings were dynamically adjusted as needed during the 24 hours in accordance with the low PEEP/$FiO_2$ table (Figure 1S).

During this decremental PEEP maneuver, PEEP was titrated simultaneously, according to the three following methods:

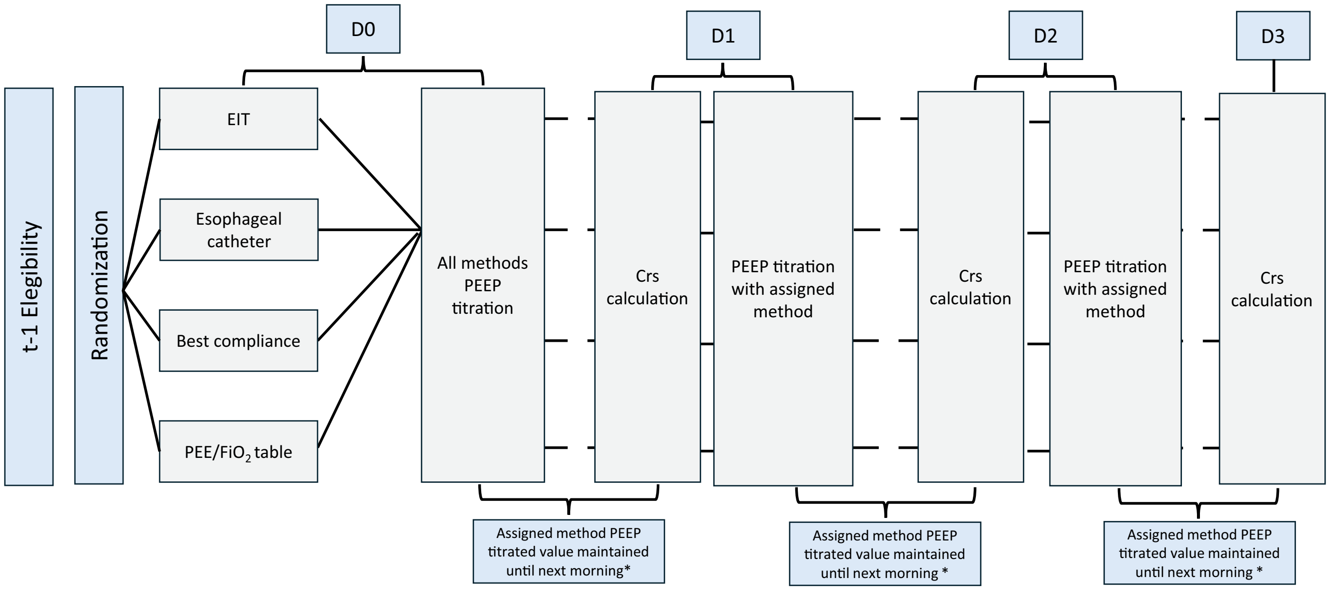


EIT - electrical impedance tomography; PEEP - positive end expiratory pressure; $FiO_2$ - fraction of inspired oxygen; Crs - compliance respiratory system.

**Figure 1S -** Overview of study procedures.

*The parameters measured in the PEEP titration remained until the following day when the Crs was initially measured with previous titrated PEEP and then a new PEEP was titrated for the next hours until the following morning, except on D3 where only the Crs was measured without a new titration

## Electrical impedance tomography

Electrical impedance tomography (EIT) assessments were taken with Enlight 1800 Timpel SA Sao Paulo Brazil, with a belt located in the 5th or 6th intercostal space (Figure 2S). Regional collapse and overdistension percentages were estimated based on the decrease of regional compliance curve calculated during the decremental PEEP trial.[2] The EIT titrated PEEP was the PEEP level corresponding to the intercept point of cumulative collapse and overdistension percentage curves, providing the best compromise between collapsed and overdistended lung.[3] If the intercept point took place between two PEEP steps, the EIT titrated PEEP would correspond to the PEEP step toward the lowest collapse percentage (Figure 3S).

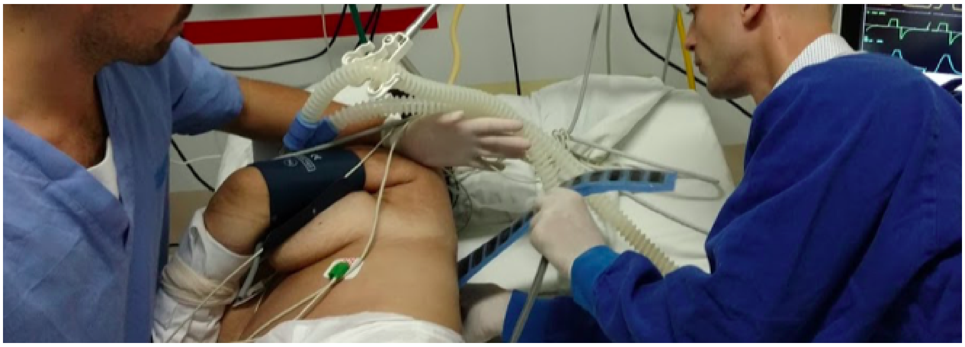

**Figure 2S -** Thoracic electrical impedance tomography belt positioning.

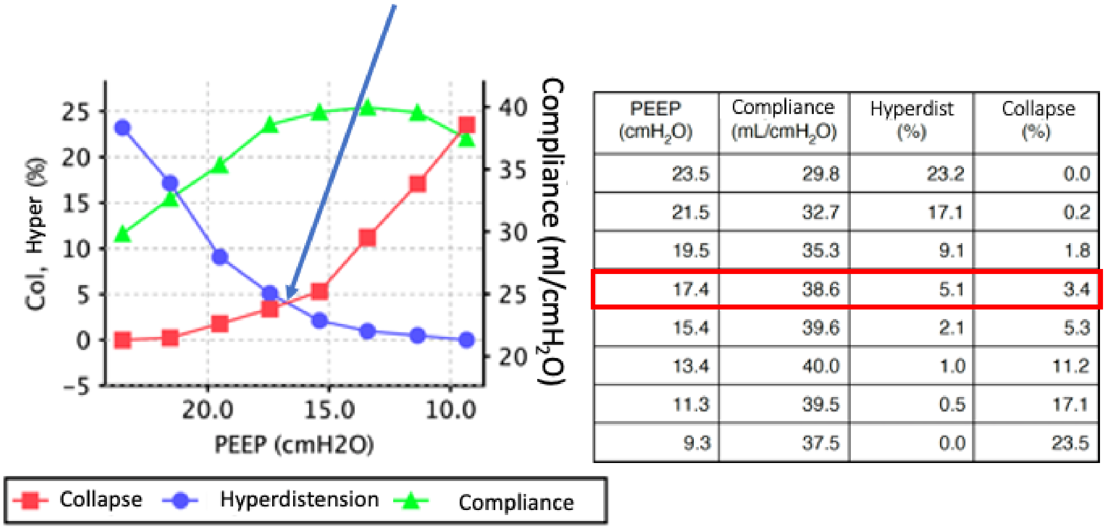


| PEEP ($cmH_2O$) | Compliance ($mL/cmH_2O$) | Hyperdist (%) | Collapse (%) |
|---|---|---|---|
| 23.5 | 29.8 | 23.2 | 0.0 |
| 21.5 | 32.7 | 17.1 | 0.2 |
| 19.5 | 35.3 | 9.1 | 1.8 |
| 17.4 | 38.6 | 5.1 | 3.4 |
| 15.4 | 39.6 | 2.1 | 5.3 |
| 13.4 | 40.0 | 1.0 | 11.2 |
| 11.3 | 39.5 | 0.5 | 17.1 |
| 9.3 | 37.5 | 0.0 | 23.5 |

PEEP - positive end expiratory pressure; Hyperdist - hyperdistention.

**Figure 3S -** Method of choosing the titrated positive pressure ventilation in electrical impedance tomography with arrow showing the point of intercept between hypertension and collapse and the values on the table in the right.

## Esophageal catheter procedure

NutriVent™ ; Seda S.p.A, Milan, Italy, was used as an esophageal catheter. The technique for insertion and measurement was performed as described before.[4,5] With the patient in a supine position and with the head of the bed elevated to 30°, a catheter (NutriVent™; Seda S.p.A, Milan, Italy was inserted through one of the nostrils with the balloon deflated. The catheter was advanced 60cm from the anterior incisors into the stomach, at which point the balloon was inflated with 0.5mL of air. Henceforward, the esophageal balloon was retracted to 40 - 45cm. The esophageal pressure position was validated daily before making any measurements to ensure accuracy.

Pes signal was measured at the catheter's proximal end by an AC1164 VC data acquisition controller with a pressure sensor capable of amplifying and digitalizing it. After adequate calibration in a U-shaped water column manometer, this signal was read in a personal computer by the software Labview, Lynx Tecnologia Eletrônica LTDA. Confirmation of the esophageal location of the catheter in the distal third of the esophagus was given by the presence of a cardiac artifact signal and/or visualization of the radiopaque markers of the device on the chest X-ray. After confirmation, complete deflation of the balloon was performed with a negative pressure of $-20cmH_2O$ using a 10mL syringe. Thereupon, we inflated the balloon with 10mL of air and removed 6.5mL, the remaining 3.5mL in the catheter. This is the mean value of balloon air volume shown in the literature to be best associated with the appropriate ratio of esophageal pressure to airway pressure (DPes/DPaw) value between 0.8 and 1.2, which indicates the balloon is appropriately located, providing a valid measurement of Pes. The ratio was measured through a maneuver with 3 to 5 manual chest wall compressions, with the patient sedated in an expiratory pause. Right after the validation of the catheter location, the Paw and Pes curves plotted against time, as well as the curve of difference between them (transpulmonary pressure (PL), were captured in the monitor. Pes at the end of inspiration (Pes insp) and expiration (Pes exp) were measured after a 2-second inspiratory or expiratory pause in the ventilator to measure inspiratory transpulmonary pressure (PL insp= Pplat – Pes insp), expiratory transpulmonary pressure (PL exp= Pplat – Pes exp) and transpulmonary driving pressure (PL insp- PL exp). These procedures are explained in figures 4S.

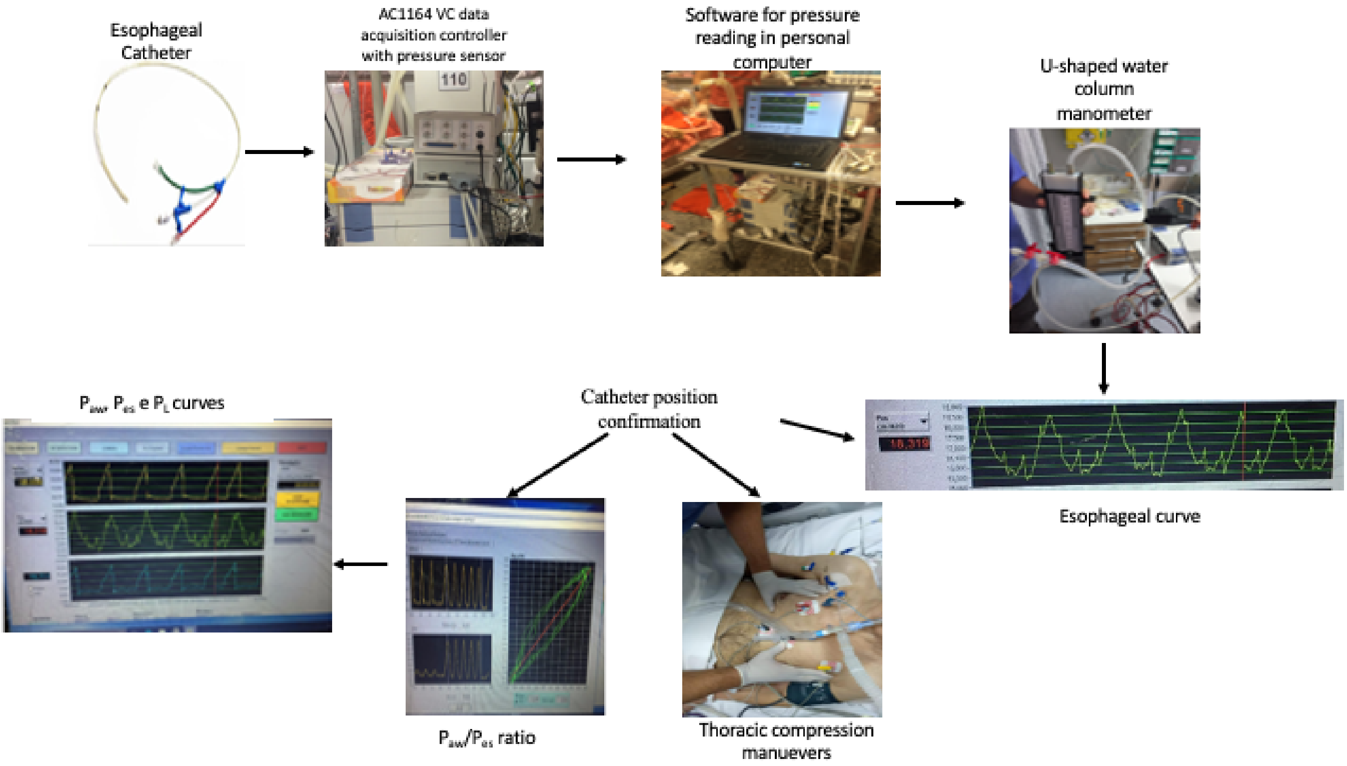


Paw - airway pressure, Pes - esophageal pressure, $P_L$ - transpulmonary pressure.

**Figure 4S -** Summary of esophageal pressure procedure starting with insertion of the catheter, reading of pressures, calibration, catheter position confirmation and airway pressure, esophageal pressure and transpulmonary pressure tracings.

The esophageal catheter titrated PEEP was the pressure that resulted in transpulmonary expiratory pressure (PLexp) > 0 and ≤ $2cmH_2O$ during the decremental PEEP maneuver. The calculation of transpulmonary pressure was done using the absolute value of esophageal pressure (direct measure).[5]

## Best respiratory system static compliance

The $C_{rs}$ value was calculated as tidal volume (in mL) divided by plateau pressure minus PEEP and was obtained for each PEEP level, from 23 to $7cmH_2O$, during the decremental PEEP maneuver after 0.5 seconds of inspiratory hold. The titrated PEEP value in this method was the pressure measured immediately before the decrease ≥ $1cmH_2O$ in $C_{rs}$. If several PEEP steps had $C_{rs}$, the highest PEEP value was the titrated PEEP selected.

From D1 to D3 after randomization, PEEP was repeatedly titrated daily only with the randomly assigned method, as described above, and maintained for 24 hours, except if SpO2 decreased to lower than 90%. In this case, PEEP was re-titrated with the assigned method.

All lung mechanics parameters, including compliance, were measured after 24 hours before new titration was done (Figure 1S). Blood gas analysis was collected 1 hour after titration.

## SECTION 5S - MECHANICAL VENTILATION PARAMETERS SET AFTER POSITIVE END EXPIRATORY PRESSURE TITRATION

Participants were ventilated in a volume-controlled mode with 4 to 6mL/kg of ideal body weight, Pplat < $30cmH_2O$, adequate RR to maintain pH > 7.30, varying up to 35 bpm, $FiO_2$ sufficient to keep peripheral $SpO_2$ > 90%. When the pH was lower than 7.30, the respiratory rates could be increased up to the limit of 35 bpm, as long as auto-PEEP levels were not higher than $2cmH_2O$. Dead-space reduction with replacement of the heat and moisture exchanger by active external humidifier, treatment of hyperthermia, adequate sedation, and neuromuscular blockade were also used to manage respiratory acidosis.

## SECTION 6S - DATA COLLECTION

Data were collected immediately before randomization (baseline), during the first PEEP titration maneuver immediately after randomization (D0) (Table 1S), 1 hour after PEEP titration on D0, and then, every morning, starting on the day after randomization (Day 1) up to Day 3, at ICU discharge or hospital discharge, and on Day 28. We collected the following variables before randomization: demographic variables, comorbidities, height, Simplified Acute Physiologic Score 3, Sequential Organ Failure Assessment variables, ventilatory parameters and arterial blood gas measurements (Tables 2S and 3S and Figures 5S and 6S), cause and duration of ARDS, and days on mechanical ventilation. Polymerase chain reaction (PCR) for influenza was collected in tracheal aspirates at the physician's discretion. Reverse transcriptase polymerase chain reaction (RT-PCR) testing for COVID-19 was ordered at the physician's discretion in March 2020.

**Table 2S -** Ventilatory and blood gas variables in baseline and from D0 to D3 between treatment groups

| Variables | Period | EIT (n = 12) | Catheter (n = 13) | Best compliance (n = 12) | PEEP table (n = 12) | Total (n = 49) | p value |
|---|---|---|---|---|---|---|---|
| Inspiratory transpulmonary pressure | Baseline | 13.1 (9.1 - 15) (n = 10) | 15.1 (13.6 - 16.2) (n = 11) | 11.9 (10.4 - 16) (n = 9) | 10.4 (8.1 - 14.2) | 13.2 (10.2 - 15.9) (n = 42) | 0.13 |
| | Median (d0 to d3) | 12.1 (8.8 - 13.8) | 12.3 (11.9 - 14) | 10.52 (8.4 - 14.4) | 10.0 (8.6 - 12.6) | 11.9 (9.0 - 14) | 0.34 |
| Expiratory transpulmonary pressure | Baseline | 0.4 (-1.9 - 1.9) (n=10) | 1.0 (0.3 - 2.6) (n = 11) | 1.8 (-0.5 - 3.9) (n = 9) | -0.2 (-3.3 - 1.0) | 0.8 (-1.2 - 2.6) (n = 42) | 0.35 |
| | Median (d0 to d3) | 0.4 (-1.4 - 2.6) | 1.2 (0.6 - 2.9) | -0.5 (-2.6 - 1.7) | -1.1 (-1.9 - 0.3) | 0.3 (-1.43 - 2.3) | 0.08 |
| Pplat titrated PEEP | Baseline | 25 (23 - 29) | 27 (26 - 30) | 29 (26 - 30) | 24 (23 - 27) | 26 (23 - 30) | 0.10 |
| | Median (d0 to d3) | 24 (22 - 27.6) | 26 (24 - 28.7) | 26 (24 - 28) | 23 (21 - 26) | 25 (22 - 28) | 0.55 |
| Driving pressure titrated PEEP | Baseline | 13 (11 - 14) | 16 (15 - 18) | 15 (12 – 19) | 13 (12 - 16) | 14 (12 - 16) | 0.06 |
| | Median (d0 to d3) | 13 (12 - 14) | 13 (12 - 17) | 13 (13 - 16) | 13 (12 - 16) | 13 (12 - 16) | 0.93 |
| Compliance titrated PEEP | Baseline | 26 (25 - 33) | 22 (18 - 25) | 25 (21 - 29) | 26 (23 - 33) | 25 (22 - 32) | 0.12 |
| | Median (d0 to d3) | 29 (24 - 33) | 25 (20 - 33) | 27 (24 - 34) | 27 (25 - 38) | 27 (23 - 35) | 0.60 |
| pH | Baseline | 7.24 (7.16 - 7.27) | 7.3 (7.26 - 7.31) | 7.38 (7.25 - 7.41) | 7.29 (7.23 - 7.33) | 7.28 (7.22 - 7.35) (n = 48) | 0.15 |
| | Median (d0 to d3) | 7.29 (7.21 - 7.33) | 7.31 (7.3 - 7.39) | 7.39 (7.33 - 7.41) | 7.36 (7.23 - 7.41) | 7.33 (7.28 - 7.4) | 0.17 |
| $PaO_2$ | Baseline | 86 (70 - 148) (n = 11) | 106 (89 - 119) (n = 12) | 112 (101 - 156) (n = 12) | 117 (108 - 139) (n = 12) | 113 (90 - 144) (n = 47) | 0.44 |
| | Median (d0 to d3) | 105 (94 - 116) (n = 12) | 115 (88 - 12) (n = 13) | 126 (108 - 140) (n = 12) | 109 (107 - 129) (n = 12) | 111 (93 - 131) (n = 49) | 0.37 |
| $PaCO_2$ | Baseline | 52 [47 - 57] (n = 11) | 58 (50 - 62) (n = 12) | 50 (45 - 59) | 47 (43 - 51) | 51 (45 - 60) (n = 47) | 0.10 |
| | Median (d0 to d3) | 58 (53 - 64) | 60 (57 - 69) | 53 (46 - 60) | 51 (48 - 54) | 56 (51 - 63) | 0.03 |
| $PaO_2/FiO_2$ | Baseline | 129 (77 - 182) | 165 (105 - 196) | 190 (152 - 211) | 192 (169 - 219) | 172 (117 – 203) | 0.07 |
| | Median (d0 to d3) | 211 (151 - 242) | 189 (163 - 236) | 259 (239 - 267.06) | 232 (183 - 244) | 226 (176 - 252) | 0.04 |
| PEEP | Baseline | 12 (10 - 14) | 10 (10 - 14) | 11 (10 - 15) | 11 (10 - 15) | 10 (10 - 14) | 0.50 |
| | Median (d0 to d3) | 10 (9 - 14) | 11 (10 - 13) | 10 (9 - 12) | 10 (9 - 12) | 10 (9 - 13) | 0.87 |

EIT - electrical impedance tomography; PEEP - positive end expiratory pressure; Pplat - plateau pressure; $PaO_2$ - arterial partial pressure of oxygen; $FiO_2$ - inspired fraction of oxygen. Results expressed as median (interquartile range).

**Table 3S -** Median titrated positive pressure ventilation in each method in D0 only

| Variable | EIT (n = 49) | Catheter (n=49) | Best compliance (n = 49) | PEEP table (n = 49) |
|---|---|---|---|---|
| PEEP | 10 (7 - 13) (n = 48) | 11 (9 - 13) (n = 46) | 11 (9 - 13) (n = 46) | 10 (10 - 14) (n = 49) |

EIT - electrical impedance tomography; PEEP - positive end expiratory pressure. Results expressed as median (interquartile range).

If the participants were discharged from the hospital before Day 28 after randomization, the investigator team contacted them or their relatives by telephone to obtain follow-up data. Mechanical ventilation parameters, fluid balance, and hemodynamic variables were measured once daily on Days 1 to 3.

## SECTION 7S - EXPLORATORY OUTCOMES

Exploratory outcomes were 28-day mortality, ventilator-free days, the occurrence of barotrauma, duration of intravenous sedatives use, neuromuscular blocking agents, vasopressors, corticosteroids, use of prone position, hospital and ICU length of stay (LOS) (Table 4S).

**Table 4S -** Exploratory outcomes analysis from Day 0 through Day 3

| Variables | EIT (n = 12) | Catheter (n = 13) | Best compliance (n = 12) | PEEP table (n = 12) | Total (n = 49) | p value* |
|---|---|---|---|---|---|---|
| Ventilator free days | 19 (14 - 23) | 17 (13 - 21) | 20 (11 - 23) | 21 (18 - 22) | 20 (13.73) | 0.70 |
| Time of sedation | 8 (5 - 12) | 10 (8 - 11) | 9 (5 - 13) | 7 (5-9) | 9 (5 - 11) | 0.7 |
| Time of neuromuscular blocking agents | 5 (3 - 6) | 4 (3 - 5) | 3 (3 - 6) | 4 (3 - 6) | 4 (3 - 6) | 0.89 |
| Time in vasopressors | 3 (2 - 5) | 2 (0 - 3) | 2 (0 - 3.5) | 3 (2 - 6) | 2 (1 - 5) | 0.42 |
| Time of corticosteroids use | 5 (1 - 16) | 4 (0 - 11) | 4 (1 - 8) | 8 (0 - 14) | 5 (0 - 12) | 0.93 |
| Hospital LOS | 20 (7 - 24) | 15 (14 - 21) | 20.5 (3 - 22) | 17 (9 - 22) | 18 (11 - 23) | 0.81 |
| ICU LOS | 13 (6 - 23) | 13 (10 - 16) | 13 (9 - 20) | 9 (8 - 15) | 12 (8 - 18) | 0.73 |
| 28-day mortality | 7/12 (58) | 4/13 (31) | 2/12 (17) | 6/12 (50) | 19/49 (39) | 0.15 |
| Barotrauma | 0/12 (0) | 0/13 (0) | 0/12 (0) | 0/12 (0) | 0/49 (0) | 1 |
| Prone position | 6/12 (50) | 9/13 (70) | 5/12 (42) | 4/12 (33) | 24/49 (49) | 0.36 |

EIT - electrical impedance tomography; PEEP - positive end expiratory pressure; LOS - length of stay; ICU - intensive care unit. *Kruskal-Wallis test; Fisher exact test. Results expressed as n/N (%) or median (interquartile range).

## SECTION 8S- PRIMARY OUTCOME

Primary outcome results with mixed effect linear regression considering an interaction between the randomization group and the time are shown in table 5S.

**Table 5S -** Primary outcome results with mixed effect linear regression considering an interaction between the randomization group and the time

| | Estimate | SE | 95%CI | p value |
|---|---|---|---|---|
| (Intercept) | 2.50 | 2.2 | (-1.73 - 6.73) | 0.26 |
| Group EIT | 0.52 | 1.93 | (-3.28 - 4.31) | 0.79 |
| Group catheter | -0.16 | 1.94 | (-3.98 - 3.66) | 0.93 |
| Group best compliance | 2.45 | 1.93 | (-1.35 - 6.24) | 0.21 |
| Day (numeric) | -1.29 | 0.62 | (-2.49 - -0.09) | 0.04 |
| Baseline compliance | 1.04 | 0.06 | (0.92 - 1.16) | < 0,01 |
| Group EIT (day) | -0.36 | 0.89 | (-2.07 - 1.36) | 0.69 |
| Group catheter (day) | 1.38 | 0.9 | (-0.35 - 3.11) | 0.13 |
| Group best compliance (day) | -0.74 | 0.89 | (-2.45 - 0.98) | 0.41 |

SE -standard error; 95%CI - 95% confidence interval; EIT - electrical impedance tomography.

Section 8S - Bland Altman analysis for agreement between the four positive end expiratory pressure titrating methods related to positive end expiratory pressure titrated and compliance respiratory system values and correlation among the PEEP levels titrated by the four methods, as well as correlation among the Crs measurements obtained using the same four methods

The Bland Altman analysis for agreement shows that the PEEP titrated by PEEP/$FiO_2$ table was 0.1cm$H_2O$ lower than PEEP titrated by the catheter with limits of agreement from -9.3 to 9.0cm$H_2O$; 0.1cm$H_2O$ higher than PEEP titrated by the best compliance with limits of agreement from -6.2 to 6.5cm$H_2O$ and 1.3cm$H_2O$ higher than PEEP titrated by EIT with limits of agreement from -4.7 to 7.4cm$H_2O$. PEEP titrated by the catheter was 0.3cm$H_2O$ higher than PEEP titrated by the best compliance with limits of agreement from -8.2 to 8.8cm$H_2O$ and 1.3cm$H_2O$ higher than PEEP titrated by EIT with limits of agreement from -6.8 to 9.5cm$H_2O$. Finally, PEEP titrated by best compliance was 1.0cm$H_2O$ higher than PEEP titrated by the EIT with limits of agreement from -3.0 to 5.1cm$H_2O$. The Bland Altman analysis for agreement shows that the Crs values derived from PEEP titrated by PEEP/$FiO_2$ table were 1.5cm$H_2O$ higher than Crs measured by the catheter with limits of agreement from -8.5 to 11.4cm$H_2O$; 2cm$H_2O$ lower than Crs measured by the best compliance with limits of agreement from -6.4 to 2.5cm$H_2O$ and 1.7cm$H_2O$ lower than Crs measured by EIT with limits of agreement from -10.0 to 6.6cm$H_2O$. Crs measured by the catheter was 3.4cm$H_2O$ lower than Crs measured by the best compliance with limits of agreement from -13.2 to 6.3cm$H_2O$ and 2.6cm$H_2O$ lower than Crs measured by EIT with limits of agreement from -12.1 to 6.9cm$H_2O$. Finally, Crs measured by best compliance was 0.8cm$H_2O$ higher than Crs measured by the EIT with limits of agreement from -2.7 to 4.4cm$H_2O$ (Figure 5).

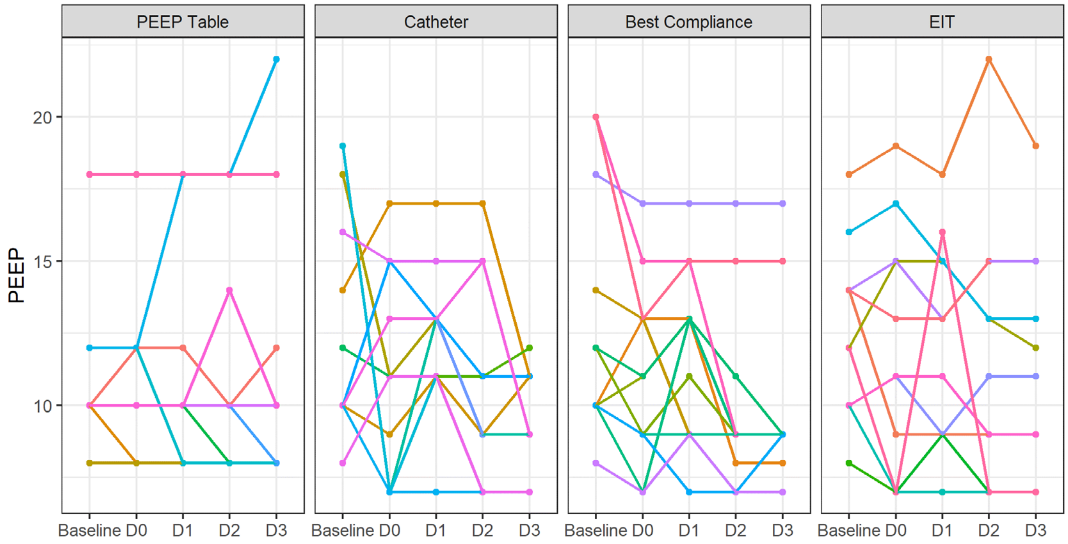


PEEP - positive end expiratory pressure; EIT - electrical impedance tomography.
**Figure 5S -** Variability of positive end expiratory pressure values individually from baseline do Day 3.

Mean $C_{rs}$ over three days increased in all groups, with greater variation in the Catheter Group, which started and ended with lower values compared to the other groups. Significant variability was observed in the individually titrated PEEP and $C_{rs}$ values, as well as in the mean titrated PEEP for each group over the course of three days (Figures 3, 5S and 6S).

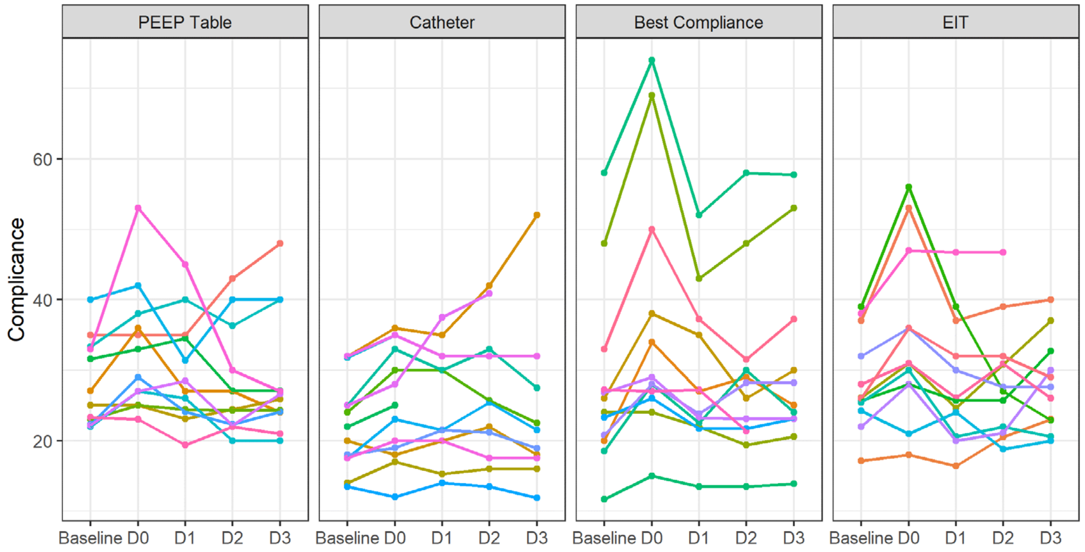


PEEP - positive end expiratory pressure; EIT - electrical impedance tomography.

**Figure 6S -** Variability of compliance curve individually from baseline do Day 3.

The correlation between PEEP titrated by the four methods varied considerably according to figure 7S.

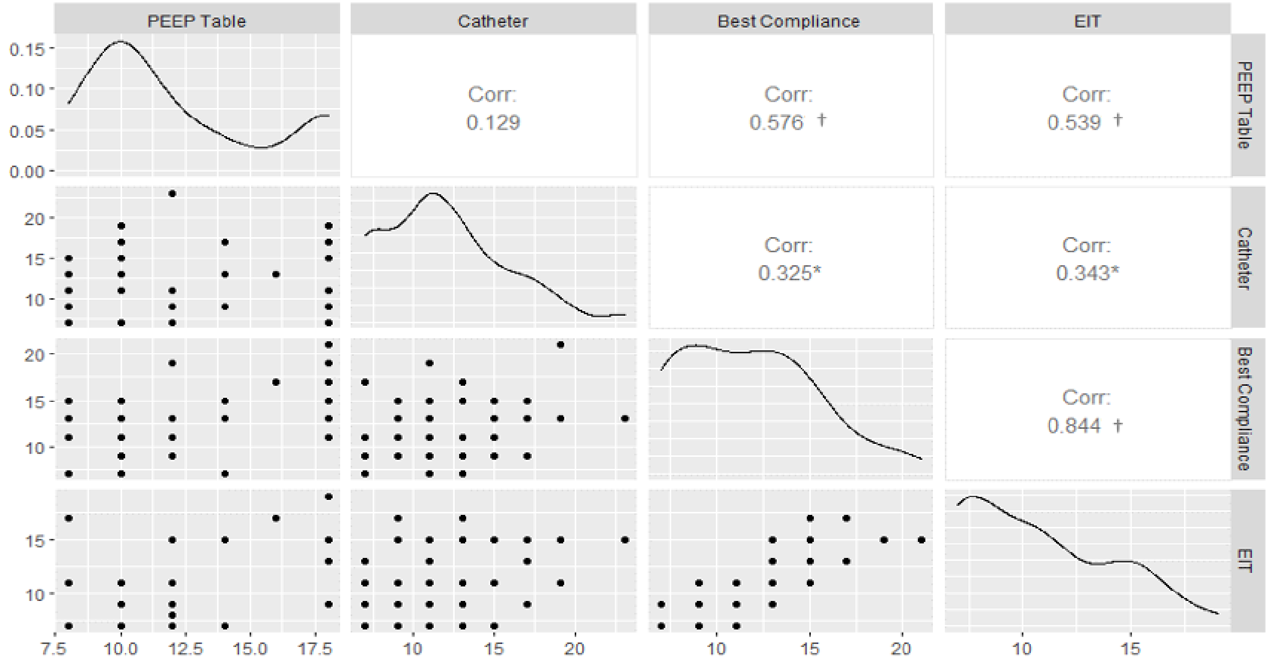


PEEP - positive end expiratory pressure; EIT - electrical impedance tomography.

**Figure 7S -** Correlation between positive end expiratory pressure titrating methods.

Pearson correlation coefficient. † p < 0,001 *p < 0,01.

However, there was a strong correlation among all methods regarding the Crs values calculated from the titrated PEEP on Day 0, as shown in figure 8S.

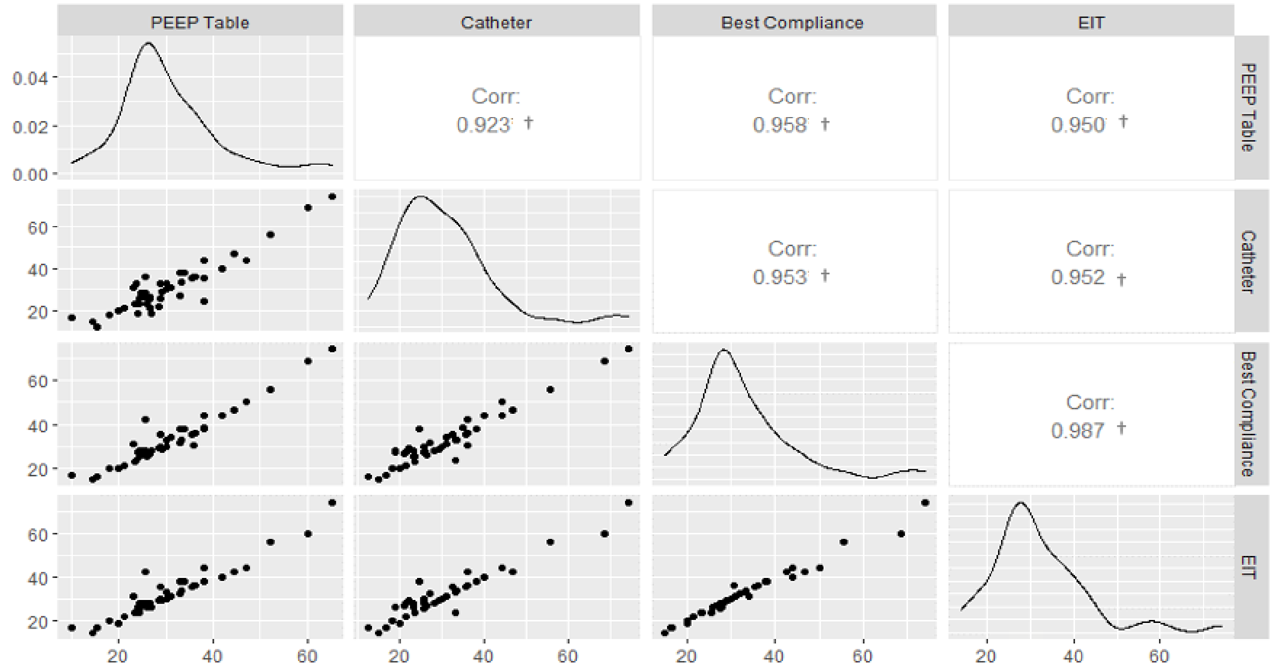


PEEP - positive end expiratory pressure; EIT - electrical impedance tomography.

**Figure 8S -** Correlation between respiratory system compliance values calculated from titrated positive end expiratory pressure values from different methods.

Pearson correlation coefficient. † p < 0,001.

## REFERENCES


1. Brower RG, Matthay MA, Morris A, Schoenfeld D, Thompson BT, Wheeler A; Acute Respiratory Distress Syndrome Network. Ventilation with lower tidal volumes as compared with traditional tidal volumes for acute lung injury and the acute respiratory distress syndrome. N Engl J Med. 2000;342(18):1301-8.
2. Costa EL, Borges JB, Melo A, Suarez-Sipmann F, Toufen C Jr, Bohm SH, et al. Bedside estimation of recruitable alveolar collapse and hyperdistension by electrical impedance tomography. Intensive Care Med. 2009;35(6):1132-7.
3. Frerichs I, Amato MB, van Kaam AH, Tingay DG, Zhao Z, Grychtol B, et al.; TREND study group. Chest electrical impedance tomography examination, data analysis, terminology, clinical use and recommendations: consensus statement of the TRanslational EIT developmeNt stuDy group. Thorax. 2017;72(1):83-93.
4. Akoumianaki E, Maggiore SM, Valenza F, Bellani G, Jubran A, Loring SH, et al.; PLUG Working Group (Acute Respiratory Failure Section of the European Society of Intensive Care Medicine). The application of esophageal pressure measurement in patients with respiratory failure. Am J Respir Crit Care Med. 2014 189(5):520-31.
5. Talmor D, Sarge T, Malhotra A, O'Donnell CR, Ritz R, Lisbon A, et al. Mechanical ventilation guided by esophageal pressure in acute lung injury. N Engl J Med. 2008;359(20):2095-104.
